# Knowledge, attitude, and practice of Nepalese residents in the prevention and control of COVID-19: A cross-sectional web-based survey

**DOI:** 10.1016/j.amsu.2022.104861

**Published:** 2022-11-12

**Authors:** Manish Rajbanshi, Sandesh Bhusal, Kiran Paudel, Bikram Poudel, Amrit Gaire, Erina Khatri, Bhoj Raj Kalauni, Biplav Aryal, Ghanashyam Sharma, Namrata Karki

**Affiliations:** a Institute of Medicine, Tribhuvan University, Maharajgunj, Kathmandu, Nepal; b Research and Development Divison, Dhulikhel Hospital, Kathmandu University, Nepal; c Nepal Health Frontiers, Tokha-5, Kathmandu, Nepal; d The Leprosy Mission, Nepal

**Keywords:** Attitude, COVID-19, Infection control, Knowledge, Nepal, Practice

## Abstract

**Background:**

Coronavirus disease 2019 (COVID-19) has caused a global public health crisis. Preventive measures to tackle the deadly virus are influenced by people's knowledge, attitude, and practice (KAP) toward COVID-19. This study aimed to assess the level of knowledge, attitude, and practice toward COVID-19 among Nepalese residents in Nepal.

**Methodology:**

A web-based cross-sectional survey was conducted among 755 Nepalese residents across all seven provinces of Nepal. The questionnaire used to determine the KAP of the participants was derived from a previous study conducted in Nepal. Descriptive analysis was done to identify the distribution of socio-economic and demographic characteristics of participants. Factors associated with residents’ KAP regarding COVID-19 were examined using Chi-square tests at the significance level of 0.05.

**Results:**

The mean age of the participants was 24.6 years. At the time of data collection, 8.2% of the participants had their families in isolation or quarantine center. In this study, 76.4%, 58.0%, and 63.6% of the participants had a good knowledge level, attitude level, and practice level respectively regarding COVID-19. Occupation and marital status were significantly associated with knowledge, attitude, and practice level. Age was significantly associated with knowledge and attitude level. Those participants who had their family members in quarantine were found to have a good level of preventive practice. The knowledge-attitude (r_ka =_ 0.184, p < 0.001), attitude-practice (r_ap_ = 0.125, p < 0.001) and knowledge-practice (r_kp =_ 0.07, p < 0.05) were positively correlated in this study.

**Conclusion:**

This study showed satisfactory awareness regarding COVID-19 among Nepalese residents. Community-based health education programs should be promoted to develop a positive attitude toward healthy practices to tackle the COVID-19 pandemic or any future health crisis.

## Highlights

• In this study, 76.4%, 58.0%, and 63.6% of the participants had a good knowledge (K), good attitude (A), and good practice (P) level respectively regarding COVID-19.
• Occupation and marital status were significantly associated with KAP of participants. Similarly, those who had their family members in quarantine were found to have a good level of preventive practice.
• This study included participants from all of the seven provinces of Nepal during the peak of the COVID-19 pandemic.

## 1 Introduction

Coronavirus disease 2019 (COVID-19) is a global public health emergency detected on December 31, 2019, in Wuhan, China [1]. Nepal is no exception to this pandemic, with overwhelming effects on its economy and healthcare system [2,3]. Following the World Health Organization (WHO) declaration of the COVID-19 outbreak, countries around the globe, including Nepal, have been leaning on response plans to respond to the pandemic and contain the virus [4].

As of July 3, 2022, 979,694 cases and 11,952 deaths of COVID-19 had been reported in the country despite adopting preventive measures like stay-at-home mandates, quarantine, and social distancing [5]. The success or failure of these preventive measures implemented by the country or concerned organizations like WHO is largely dependent on public behavior [6]. Studies conducted during the early phase of the COVID-19 pandemic have shown that public knowledge, attitude, and practice toward the virus are important in tackling the pandemic [7]. By assessing public awareness and knowledge about COVID-19, deeper insights into existing public perception and practices can be gained, thereby helping to identify attributes that influence the public in adopting healthy practices and responsive behavior [8].

Several studies have suggested a need to investigate the KAP towards COVID-19 among residents of low socioeconomic status countries like Nepal to encourage an optimistic attitude and maintain safe practices [9]. Assessing the public's knowledge, attitude and practice are also important in identifying gaps and strengthening ongoing preventive efforts.

Therefore, this study aims to assess the KAP toward COVID-19 among the general public of Nepal. These findings are anticipated to aid authorities in better organizing public awareness programs to eliminate COVID-19-related myths and malpractices, potentially resulting in the disease being curtailed.

## 2 Materials and methodology

### 2.1 Study design and setting

A web-based cross-sectional study was carried out in all of the seven provinces in Nepal. Data were collected from the 10th to the 30th of December 2020.

### 2.2 Study population and sampling

The study population was Nepalese residents (aged 18 years or above) across all the seven provinces of Nepal during COVID-19 pandemic. A convenience sampling procedure was adopted to collect the data. A total of 755 participants were recruited in the survey.

### 2.3 Measures

The study questionnaire was derived from the study conducted by Hussain et al. in the early phase of the COVID-19 pandemic in Nepal [10]. The online questionnaire consists of two sections, first is related to the socio-economic and demographic characteristics of the participants that includes; sex (male/female), age (in years), marital status (married/unmarried), occupation (health/non-health sector), family monthly income [≤ NRs. 40,000 (USD 310) and > NRs. 40,000 (USD 310)], and family member in quarantine or isolation (Yes/No).

The second section consists of questions on KAP on COVID-19. Altogether there were a total of 28 questions including 15 on knowledge, 6 on attitude, and 7 on practice. Knowledge-related questions were regarding cause, symptoms, transmission mode, incubation period, vulnerable group, and preventive measures. Attitude-related questions were regarding control of COVID-19, alcohol drinking as a protective measure, self-protection, and effectiveness of lockdown. Similarly, practice-related questions were regarding mask use, hand washing, social distancing, and following lockdown. Evaluation of knowledge, attitude, and practice was done by assigning ‘1’ for positive/correct answers and ‘0’ for negative/incorrect answers. A score of 13 or above was considered as a good knowledge level while respondents' scores of 5 or above were considered good attitude levels. Similarly, a score of 5 or above was considered a good practice level in our study.

This study has been reported in line with the STROCSS criteria [11].

### 2.4 Data collection

Online Google forms questionnaire was administered to the participants through social media platforms to collect the data. Single response from each student was ensured via Google Forms by choosing ‘Limit to 1 response’.

### 2.5 Data management and analysis

Data from the Google forms were automatically recorded in Google sheets. All the collected information was systematically compiled, coded, checked, and edited before exporting to Statistical Package for the Social Sciences (SPSS) version 20 (IBM) for analysis. The respondents' socio-economic and demographic characteristics were described using frequencies and percentages. Chi-square tests were performed to establish the association of KAP and demographic variables as appropriate. Pearson coefficient of correlation was used to determine the relationship between knowledge-attitude, attitude-practice, and knowledge-practice. The statistical significance was set at p-value < 0.05.

### 2.6 Ethical approval

The approval for this study was taken from the Institutional Review Committee, of the Institute of Medicine [Ref no: 109(6-1) E2077/078]. Study objectives were explained in the Google forms, and e-informed consent was taken from all the participants before the data collection (UIN no. researchregistry8325).

## 3 Results

### 3.1 Characteristics of the study participants

A total of 755 Nepalese residents participated in the study. An almost equal proportion of males (50.6%) and females (49.4%) participated in the study. The majority of the participants (68.6%) belonged to the age group below 25 years. Half of the participants were Brahmin/Chhetri (49.4%) ethnic group followed by Janajati (30.6%). In this study, more than one third of the participants were from Province One (38%) followed by Bagmati Province (23.6%) (Table 1).

**Table 1:** Characteristics of the study participants.

Characteristics	Numbers (n)	Percentage (%)
Sex		
Male	382	50.6
Female	373	49.4
**Age group (in years)**		
Below 25	518	68.6
25 or above	237	31.4
**Ethnicity**		
Brahmin/Chhetri	373	49.4
Janajati	231	30.6
Madhesi	121	16.0
Others	30	4.0
**Religion**		
Hindu	691	91.5
Others	64	8.5
**Marital status**		
Married	128	17.0
Umarried	627	83.0
**Occupation**		
Health sector	315	41.7
Non-Health sector	440	58.3
**Province**		
Province no. 1	287	38.0
Madhesh Province	98	13.0
Bagmati Province	178	23.6
Gandaki Province	45	6.0
Lumbini Province	82	10.9
Karnali Province	14	1.9
Sudurpaschim Province	51	6.8
**Monthly income of the family**		
Below or equal to NRs.40,000 (USD 310)	205	27.2
Above NRs.40,000 (USD 310)	550	72.8
**Family members in quarantine or isolation**		
Yes	62	8.2
No	693	91.8

### 3.2 Factors associated with knowledge of COVID-19 among the participants

Table 2 shows that 76.4% of the Nepalese residents demonstrated a good knowledge level of COVID-19. The level of knowledge is significantly associated with age (OR = 1.49, CI: 1.05–2.12), sex (OR = 1.46, CI: 1.04–2.06), marital status (OR = 1.84, CI: 1.22–2.78), occupation (OR = 3.40, CI:2.29–5.04) and monthly income of the family (OR = 1.50, CI: 1.0–2.24).

**Table 2:** Factors associated with knowledge of COVID-19 among the participants.

Characteristics	Level of knowledge		p - value	Odds Ratio (95% CI)
	**Good n (%)**	**Poor n (%)**		
**Level of Knowledge**	577 (76.4)	187 (23.6)		
**Sex**				
Female	298 (79.9)	75 (20.1)	0.03	**1.46 (1.04–2.06)**
Male	279 (73.0)	103 (27.0)		
**Age**				
Below 25	408 (78.8)	110 (21.2)	0.02	**1.49 (1.05–2.12)**
25 or above	169 (71.3)	68 (28.7)		
**Marital status**				
Unmarried	492 (78.5)	135 (23.6)	<0.001	**1.84 (1.22–2.78)**
Married	85 (66.4)	43 (33.6)		
**Occupation**				
Health sector	277 (87.9)	38 (12.1)	<0.001	**3.40 (2.29–5.04)**
Non- Health sector	300 (68.2)	140 (31.8)		
**Monthly income of the family**				
Above NRs. 40,000 (USD 310)	410 (74.5)	140 (25.5)	0.04	**1.5 (1.0–2.24)**
Below or equal to NRs. 40,000 (USD 310)	167 (81.5)	38 (18.5)		
**Family member in quarantine or isolation**				
Yes	49 (7.0)	13 (21.0)	0.64	1.17 (0.62–2.25)
No	528 (76.2)	165 (23.8)		

### 3.3 Factors associated with an attitude of participants towards COVID-19

In this study, 58% of the participants had a positive attitude level towards COVID-19. The attitude level of participants towards COVID-19 was found to be significant with age (OR = 1.89, CI: 1.38–2.58), marital status (OR = 1.72, CI: 1.17–2.52), and occupation (OR = 1.37, CI: 1.02–1.85) (Table 3).

**Table 3:** Factors associated with an attitude toward COVID-19 among the participants.

Characteristics	Level of attitude		p-value	Odds Ratio (95% CI)
	Good n (%)	Poor n (%)		
**Level of Attitude**	438 (58.0)	317 (42.0)		
**Sex**				
Female	227 (60.9)	146 (39.1)	1.22	1.2 (0.94–1.68)
Male	211 (55.2)	171 (44.8)		
**Age in years**				
Below 25	326 (62.9)	192 (37.1)	<0.001	**1.89 (1.38–2.58)**
25 or above	112 (47.3)	125 (52.7)		
**Marital status**				
Unmarried	378 (60.3)	249 (39.7)	<0.001	**1.72 (1.17–2.52)**
Married	60 (46.9)	68 (53.1)		
**Occupation**				
Health sector	197 (62.5)	118 (37.5)	0.03	**1.37 (1.02–1.85)**
Non- Health sector	241 (54.8)	199 (45.2)		
**Monthly income of the family**				
Below or equal to NRs. 40,000 (USD 310)	112 (54.6)	93 (45.4)	0.28	0.82 (0.59–1.14)
Above NRs.40,000 (USD 310)	326 (59.3)	224 (40.7)		
**Family member in quarantine or isolation**				
Yes	41 (66.1)	21 (33.9)	0.18	1.45 (0.84–2.51)
No	397 (57.3)	296 (42.7)		

### 3.4 Factors associated with the practice of participants toward COVID-19

In this study, almost two-thirds (63.6%) of the respondents demonstrated a good practice level towards COVID-19. Marital status (OR = 1.62, CI: 1.10–2.39), occupation (OR = 1.06, CI: 1.18–2.17) and family member in quarantine/isolation (OR = 0.58, CI: 0.34–0.98) were significantly associated to the practice level. (Table 4)

**Table 4:** Factors associated with the practice of COVID-19 among the participants.

Characteristics	Level of practice		p-value	Odds Ratio (95% CI)
	Good n (%)	Poor n (%)		
**Level of practice**	480 (63.6)	275 (36.4)		
**Sex**				
Male	232 (60.7)	150 (39.3)	0.10	1.28 (0.95–1.72)
Female	248 (66.5)	125 (33.5)		
**Age**				
Below 25 years	335 (64.7)	183 (35.3)	0.37	1.16 (0.84–1.59)
25 or above	145 (61.2)	92 (38.8)		
**Marital status**				
Unmarried	411 (65.6)	216 (36.4)	0.01	**1.62 (1.10–2.39)**
Married	69 (53.9)	59 (46.1)		
**Occupation**				
Health	220 (69.8)	95 (30.2)	<0.001	**1.60 (1.18–2.17)**
Non- Health	260 (59.1)	180 (40.9)		
**Monthly income of the family**				
Below or equal to NRs. 40,000	132 (64.4)	73 (35.6)	0.79	1.05 (0.75–1.46)
Above NRs.40,000	348 (63.3)	202 (202)		
**Family member in quarantine or isolation**				
No	448 (64.6)	245 (35.4)	0.04	**1.72 (1.02–2.94)**
Yes	32 (51.6)	30 (48.4)		

### 3.5 Correlation between knowledge, attitude, and practice of COVID-19

Table 5 shows that knowledge and attitude were positively correlated (r = 0.18) at <0.001 level of significance. While attitude and practice were positively correlated (r = 0.12) at the significance level of <0.001. Similarly, knowledge and practice were positively (r = 0.07) correlated at the significance level of <0.05.

**Table 5:** Correlation between knowledge, attitude, and practice of COVID-19.

		Knowledge	Attitude	Practice
**Knowledge**	Pearson Correlation coefficient	1	0.18**	0.07*
**Attitude**	Pearson Correlation coefficient	0.18**	1	0.12**
**Practice**	Pearson Correlation coefficient	0.07*	0.12**	1

** p-value less than 0.001.

* p-value less than 0.05.

## 4 Discussion

This study showed that 76.4% of the Nepalese residents had a good knowledge level regarding COVID-19, which is consistent with the findings of previous studies conducted in Malaysia [12], Ethiopia [13], Saudi-Arabia [14], and Jordan [15]. This study reported a better knowledge level among Nepalese residents compared to studies conducted in Syria [16] and Thailand [17].

Participants in the lower age group (<25 years) had higher odds of having good knowledge and attitude regarding COVID-19 compared to participants in the higher age group. This is consistent with the findings of the study conducted in Nepal [4], and China [9] indicating a lower age group with a good knowledge score. This higher knowledge level among the lower age group might be due to the increased use of social media platforms during the lockdown period [15,18]. Participants belonging to the younger age group might have gained awareness of COVID-19 via television, internet, and other online media and platforms.

Females had 1.46 times higher odds of having a good knowledge level compared to their male counterparts. Studies conducted in Jordan [15], Saudi Arabia [14], and China [9] showed similar results as in our study. The finding of significantly lower knowledge scores among males is in line with previous studies reporting men are less health-conscious and more likely to engage in risk-taking behavior [19,20]. In contrast to our study males demonstrated higher knowledge levels in a similar study conducted in Ethiopia [8]. In our study, unmarried participants had higher odds of having a good knowledge, attitude, and practice level towards COVID-19. The possible reason behind this could be unmarried respondents might have spent more time on social media and benefited from the different COVID-19-related information spread during the period. Another reason might be the unmarried population are usually involved in academic institution keep updating themselves on health information. In contrast to our finding, study conducted by Akalu et al. [10] showed higher knowledge among the married population.

In our study, participants belonging to the health sector had significantly higher odds of having good knowledge, attitude, and practice toward COVID-19. The findings of previous studies conducted in Nepal [10], Vietnam [21], and China [22,23] are consistent with our findings reflecting respondents from a health background had good knowledge scores. It may be due to health professionals being in an advantageous position to access and assimilate information on COVID‐19 prevention and control [24]. This study showed knowledge level of a family with a higher income had greater odds of having good knowledge in comparison to a family with a lower income. This finding is supported by studies conducted in Syria [14], Pakistan [18], and Southern Ethiopia [8] where higher earners were more knowledgeable about COVID-19. People with higher socioeconomic levels are expected to have higher education levels and access to health information, this could be the reason behind higher knowledge of COVID-19 among those populations.

This study showed a better attitude level among Nepalese residents than the previous study conducted in Nepal [4], Ethiopia [13], and China [22]. These studies were conducted during the early phase of the COVID-19 pandemic, in that period information regarding COVID-19 was not accessible and available to the general population. So, people became more aware of COVID-19 and had a positive attitude level during the late period of the outbreak.

Almost 64% of the participants showed a good practice level in our study which is better than the study conducted among residents of Ethiopia [13]. It may be that the residents with good knowledge and a good attitude are more likely to follow good practices. Knowledge is the main modifier to bringing a positive attitude and then positive practice toward health among the public. Our study found better practice levels among the family having COVID-19 infected members. It might be that the family members are always more aware and conscious of transmission to prevent infection among the other family members.

This study showed a significant positive correlation between knowledge-attitude, knowledge-practice, and attitude-practice supporting the findings of similar studies conducted in Nepal [4], Bangladesh [25], and Syria [16]. A correlation between knowledge and practice was found to be weak, but it is hoped that it will lead to uncovering the cause of community disobedience in preventing COVID-19 transmission.

## 5 Strengths and limitations

This study included participants from all seven provinces of Nepal with a relatively bigger sample size (n = 755). Another strength of this study is that it was conducted during the peak of the COVID-19 pandemic in Nepal.

Despite its strength, this study has a few limitations. Firstly, all the measurements in this study were based on self-reports, which may have been prone to response and information bias. Secondly, this study was cross-sectional and, therefore, cannot demonstrate causality between the variables. Lastly, as this was a web-based survey, poor internet connection might have discouraged some participants to fill out the online questionnaire.

## 6 Conclusion

This study revealed a satisfactory level of knowledge, attitude, and practice toward COVID-19 during the pandemic. Based on these findings targeted community awareness intervention programs and effective health education that are aimed at improving knowledge, attitude, and practice toward disease outbreaks during the earliest phase to prevent further transmission might be useful. The findings may help local health authorities and policymakers to identify the target populations to conduct awareness programs in a future infectious disease outbreak. Collaborative efforts between health services providers, the ministry of health and population, local governments, development partners, and the media should be implement as an effective tools that increase the KAP of individuals and households regarding infectious diseases like COVID-19.

## Ethical approval

The approval for this study was taken from the Institutional Review Committee, of Institute of Medicine (Ref no: 109(6-1) E2077/078. Study objectives were explained in the Google forms, and e-informed consent was taken from all the participants before the data collection.

## Sources of funding

No funding was received for the study.

## Author contribution

MR conceived the study, administered the project, conducted formal analysis, and wrote the first draft of the manuscript. SB and KP contributed in the methodology, formal analysis, writing original draft, reviewing, and editing. BP, EK, BRK, BA, AG, and GS wrote the first draft of the manuscript. NK supervised the whole study. All authors reviewed and approved the final version of the manuscript.

## Consent

Study objectives were explained in the Google forms, and e-informed consent was taken from all the participants before the data collection.

## Registration of research studies

1. Name of the registry: None.

2. Unique Identifying number or registration ID: None.

3. Hyperlink to your specific registration (must be publicly accessible and will be checked):

## Guarantor

Sandesh Bhusal.

## Provenance and peer review

Not commissioned, externally peer-reviewed.

## Declaration of competing interest

Authors have no conflict of interest to declare.
